# Visualization of the Stimuli-responsive Surface Behavior of Functionalized Wood Material by Chemical Force Microscopy

**DOI:** 10.1038/s41598-019-54664-3

**Published:** 2019-12-06

**Authors:** Claudia Gusenbauer, Etienne Cabane, Notburga Gierlinger, Jérôme Colson, Johannes Konnerth

**Affiliations:** 1 BOKU - University of Natural Resources and Life Sciences Vienna, Department of Materials Sciences and Process Engineering, Institute of Wood Technology and Renewable Materials, Konrad-Lorenz-Straße 24, 3430 Tulln, Austria; 2 ETH Zürich, Institute for Building Materials, Stefano-Franscini-Platz 3, 8093 Zürich, Switzerland; 3 EMPA – Swiss Federal Laboratories for Materials Science and Technology, Überlandstrasse 129, 8600 Dübendorf, Switzerland; 4 BOKU - University of Natural Resources and Life Sciences Vienna, Department of Nanobiotechnology, Institute of Biophysics, Muthgasse 11, 1190 Vienna, Austria

**Keywords:** Structural materials, Cell wall, Atomic force microscopy

## Abstract

The hierarchical and porous wood structure provides a stable scaffold to design functionalized lignocellulosic materials with extended properties by chemical modification techniques. However, proper nanoscale characterization methods for these novel materials are needed to confirm the presence of the added functionality and to locate the introduced functional groups with high spatial resolution. Chemical force microscopy is a suitable characterization method to distinguish chemical surface characteristics by scanning the samples surface with a functionalized tip. We report the application of this nanotechnology method on both, unmodified and functionalized wood samples to confirm the thermo-responsive behavior of poly(N-isopropylacrylamide) (PNIPAM) modified spruce wood. By performing force measurements on ultra-microtomed surfaces, adhesion force differences on the analysed structure are monitored and reveal the location and functionality of introduced functional groups. The modified samples are scanned below and above their lower critical solution temperature with a hydrophobic tip in aqueous media to observe adhesion changes. Additionally, confocal Raman microscopy support the chemical force microscopy measurements by revealing the success of the modification and the distribution of PNIPAM across the sample cross-sections. The results show that PNIPAM is mainly located in wood cell wall areas close to the lumen in early- and transitionwood.

## Introduction

**Figure 1:**
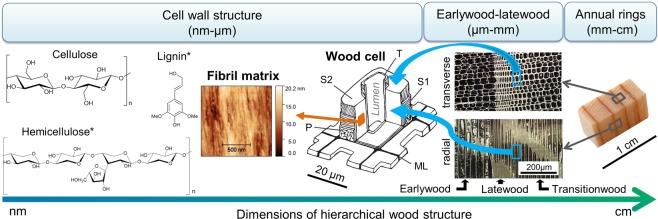
Visualization of the structure of spruce wood at different hierarchical levels (T = tertiary wall, S2/S1 = secondary wall, P = primary wall, ML = middle lamella). Cellulose chains are embedded in a lignin and hemicellulose scaffold and form a fibrillary matrix (Ø microfibrils < 35 nm)^2^. They build up the different wood cell wall layers, which vary in thickness, fibril orientation and distribution of their components^2^. (*here one exemplary precursor of the heterogeneous lignin macromolecule and one exemplary hemicellulose molecule are selected).

Wood is a porous, naturally organized and hierarchical material with unique structural properties^1^. One hierarchical level is represented by the well-known annual rings, which are formed by thicker cell walls in the latewood area and thinner cell walls in the earlywood area. At the microscopic hierarchical level, cell walls with diameters of several µm are organized by cellulose microfibrils with diameters less than 35 nm^2^, which are embedded in a lignin/hemicellulose matrix. The orientation of these fibrils varies depending on their location within the cell walls (Fig. 1). When analyzing the wood structure, two main domains can be distinguished: cell walls, which are connected by a layer called the middle lamella, and lumina, which are hollow spaces in the interior of the cell^3^. This porous framework, combined with various chemical modification techniques and state of the art nanotechnology approaches, can be utilized for the design of functional renewable nanoscale materials^4^. The applied methods target on the one hand at overcoming undesired wood properties like swelling or wood degradation and on the other hand at introducing entirely new characteristics^5–7^. Several chemical modification techniques were developed for this purpose by bonding chemical groups to the wood cell wall components^8–10^.

One specific modification technique to influence the wood surface behavior is based on the polymerization of wood cell walls with PNIPAM (poly(N-isopropylacrylamide))^11,12^. PNIPAM is a well-known stimuli-responsive polymer, whose thermal behavior in aqueous media was first characterized in the late 1960’s: it reversibly transforms from a relatively hydrophilic to a more hydrophobic state at a lower critical solution temperature (LCST) of 32 °C^13^. When modifying the inner wood surface with PNIPAM polymer chains, it should be possible to transfer the PNIPAM-inherent characteristics to the wood materials. As a result, modified wood structures should be able to change their inner surface property from a hydrophobic to a hydrophilic behavior, depending on the surrounding temperature. Before thinking about very tangible applications of such a porous material exhibiting a high internal surface area, spatial resolution characterization of the functionalized wood sections is important for a better understanding of the modification process. Moreover, insights regarding the location and character of the introduced functionality can be gained in this way.

To analyze nano- and microstructures of the samples scaffold, scanning electron microscopy and atomic force microscopy are methods of choice since they are able to track structural changes with high resolution. They however lack chemical sensitivity. To reveal information about the chemical composition of the material, e.g. infrared spectroscopy and energy-dispersive X-ray spectroscopy could be applied, but these methods lack spatial resolution. Confocal Raman spectroscopy would be able to provide structural and chemical information, but the spatial resolution is limited by the diffraction of light^14^. Atomic force microscopy-based infrared spectroscopy represents a method, which is able to provide topographical and chemical maps at the nanometer scale simultaneously. With this technique, the location of the functionalized wood structures might be revealed, but the temperature driven surface functionality could just be assumed since an aqueous environment is needed to activate the thermo-responsive functionality. To obtain these two fundamental properties simultaneously, i.e. the local distribution of the functionalized areas (up to ~30 nm) and the functionality of the surface in the corresponding environment (e.g. water), we propose to use chemical force microscopy (CFM)^15–18^.

CFM is an analyzing tool for surface properties, which is able to map functional groups on a flat surface. This technique is an extension of atomic force microscopy (AFM) with spatially resolved chemical sensitivity enabled by well-defined functionalization of the scanning tips^19–21^. Since its development about twenty-five years ago, CFM has become a well-established technique in biological and chemical sciences, being able to perform in different gaseous or liquid environments (e.g. buffer solutions)^22^. In the field of material sciences, CFM served as a characterization tool to analyze the nanoscale chemical distribution of functionalized polymers or the stimuli-responsive behavior of adhesive copolymers^23,24^. Furthermore, it allowed the measurement of adhesion properties of functionalized tips on wood to better understand mechanical properties of wood-plastic composites^25^. Additionally, adhesion force measurements provided an insight into the interaction of polylactic acid (PLA) and lignocellulosic fibers and allowed identifying surface polarity changes on bulk wood^26,27^. The application of this method could be expanded for analyzing functionalized lignocellulosic matrices, as there is a need for understanding and targeting modification techniques for novel high-tech wood materials.

We hypothesize that CFM can be applied to visualize the thermo-responsive behavior of PNIPAM modified cell wall areas on functionalized wood structures. The functionalization methods were applied on spruce wood, since the structure of this type of wood is more homogenous in comparison to other wood species. The changes in adhesion properties shall be detected by CFM on ultra-microtomed surfaces since this method offers the possibility to distinguish material properties chemically at the nanoscale^28^. In the present study, (1) we performed CFM experiments on rather rough wood surfaces with height changes up to 4 µm within the scanning area of 50 µm and temperatures up to 40 °C; (2) we measured the adhesion changes between a hydrophobic tip on a swollen wood surface in liquid environments; (3) we demonstrated that PNIPAM-modified wood cell wall areas change their surface behavior when exposed to different temperatures. This allows us verifying the reversible phase transition occurring on the modified wood surface, showing a temperature-driven adhesion switch between hydrophilic and hydrophobic states. Complementarily, we used confocal Raman spectroscopy to verify and detect the modified areas of PNIPAM-treated wood cells over larger samples cross-sections. Raman experiments revealed the location of the introduced functional groups by analyzing inelastic scattering of a light source. CFM experiments confirmed the temperature depending properties of PNIPAM-modified areas by analyzing the direct interactions of a hydrophobic tip on the samples surface. We therefore present an approach for characterizing functionalized wood materials at the nanoscale, which should fill the gap in the wood characterization techniques regarding high-resolution imaging together with chemical mapping.

## Results and Discussion

### Distribution of modification components by Raman microscopy

**Figure 2:**
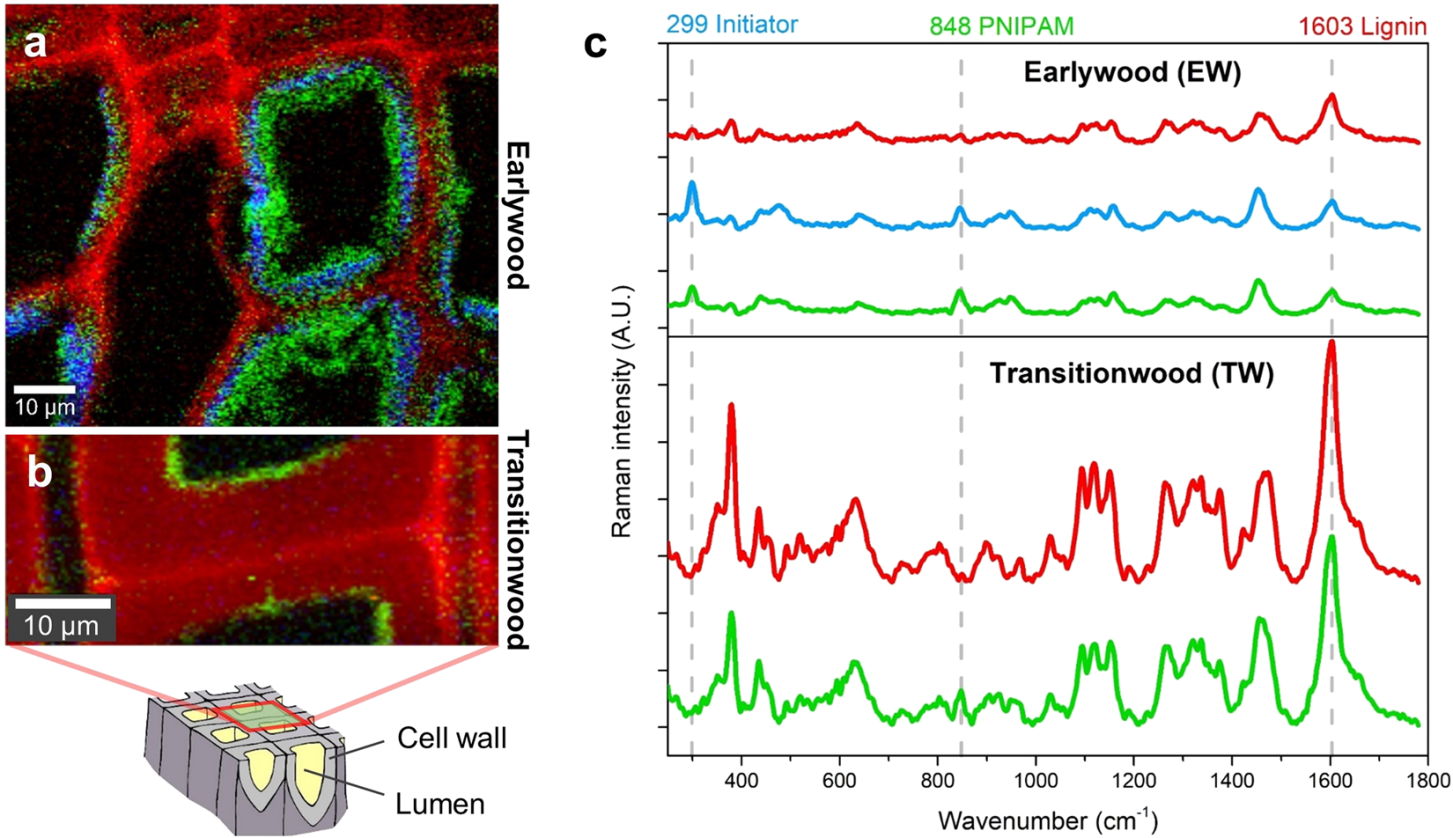
PNIPAM modified wood cell walls were analyzed with confocal Raman microscopy. (**a**,**b**) While in earlywood (EW) typical bands of the polymerization initiator were found at 299 cm^−1^, the PNIPAM assigned bands were found in EW and transitionwood (TW) at 848 cm^−1^. Integrating these two bands resulted in false color images revealing the functionalized areas within the wood cell wall cross-section (blue = initiator, green = PNIPAM, red = wood based on lignin band integration at 1603 cm^−1^) in earlywood area and transitionwood. (**c**) Average spectra of the identified chemically different regions in EW and TW were extracted.

The success of the wood functionalization process was verified by confocal Raman microscopy. Regions in which the initiator treatment was successful, showed a characteristic band at 299 cm^−1^, whereas the PNIPAM could be followed by a marker band at 848 cm^−1^ (Fig. 2c). These two bands were integrated to visualize the initiator (blue) and/or PNIPAM (green) modification in context with the wood cells (red, based on integration of lignin marker band at 1603 cm^−1^) in earlywood (Fig. 2a) and transitionwood wood (Fig. 2b).

It has to be considered that chemical treatments of wood matrices stand out for their inhomogeneous distribution over the wood cross-section due to the unique structure of its elements^29^. This property was also observed at the PNIPAM modified wood with Raman spectroscopy by overview scans (not shown). As seen in Fig. 2a,b, PNIPAM polymers were found to be heterogeneously distributed over the analyzed wood cross section area. The degree of modification of thin earlywood cell walls with large lumina was much higher and thick PNIPAM modified areas were visible (penetration depth 5 µm, green and blue area, Fig. 2a). In contrast, the polymerization at thicker transition cell walls took place just on areas close to the lumen (penetration depth 1–2 µm, green area, Fig. 2b). Most latewood cell walls showed no modification at all. The irregular distribution of the polymer derives from the nature and the function of the different wood cells. Earlywood cells are thin-walled with lumen diameters up to 30 µm. Therefore, their function is to conduct and transport water together with nutrients within the tree. Compared to these cells, latewood cells are thick-walled with small lumen diameter and their function is to provide strength^30^. Transitionwood cells show intermediate properties, in between late and earlywood cells characteristics. Thus, the transport of the functionalization chemicals is easier in the earlywood cells, which leads to a higher degree of functionalization.

The information gained with Raman spectroscopy supported the CFM measurements with the following findings: (1) PNIPAM was successfully introduced into the wood cell walls and (2) the PNIPAM modification degree increases from latewood to transitionwood to earlywood. As the functionalization made the wood cubes more brittle, the thin earlywood cell walls were therefore easily damaged while the preparation process. All further CFM tests were conducted on transitionwood cell walls. These wood cells possess the required cell wall thickness, stability and degree of modification.

### CFM on modified and native wood cell wall surfaces

**Figure 3:**
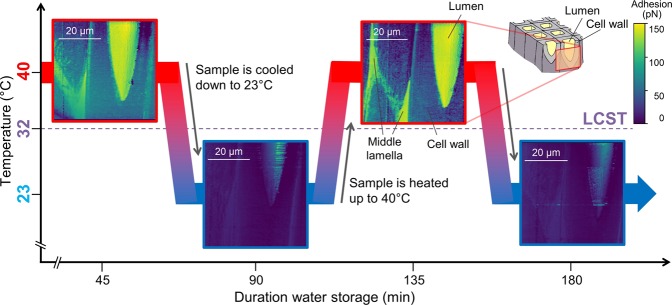
Representative CFM adhesion and topography maps of four subsequent measurements at two different temperatures of the same PNIPAM modified wood cell. The maps were obtained with a CH_3_-terminated AFM tip. Areas close to the lumen and the middle lamella showed higher adhesion to the hydrophobic tip resulting in yellowish color at 40 °C and indicate switchable adhesion forces.

To prove the thermo-responsive function of PNIPAM modified wood cell walls with high-resolution imaging, the direct interaction of a hydrophobic AFM tip on functionalized cell wall areas was analyzed using the CFM principle at two temperatures. For this purpose, the ultra-microtomed wood sample was placed on the AFM fluid cell, which was then put on the AFM heating stage. After carefully filling up the fluid cell with MilliQ water, the sample was allowed to swell for 30 min before starting the CFM measurements. As the wood cell wall structure varies naturally regarding its thickness and represents a rather rough and heterogeneous surface for conducting CFM experiments, we aimed at analyzing the same cell wall position to keep the roughness influence constant. Subsequently, PeakForce QNM maps at the radial section of a transitionwood cell wall were acquired at a water temperature of 23 °C with a hydrophobic tip. Then the temperature of the heating stage was set to 40 °C and the sample volume started to expand for some micrometers due to the temperature variation^31^. Therefore, the step motor had to be adjusted by moving up. As thermal expansion of wood is anisotropic and the thermally induced movement does therefore not only change in z-direction but also the x- and y-directions, the area of interest was traced by offsetting the x- and y-positions. Offsets up to 20 µm were necessary. The observed swelling behavior in x-, y- and z-directions belongs to the most challenging properties of wood for performing measurements in fluids, since wood is a hygroscopic material and binds water to its structure^31^. As the structure and texture of each wood sample differ naturally, the time until the samples dimensions became stable was varying. After roughly 10 min, CFM maps could be acquired at 40 °C. At this temperature, specific areas close to inner lumen surface and at the middle lamella showed a stronger adhesion towards the hydrophobic tip resulting in brighter color in adhesion maps as seen in Fig. 3. In contrast, most parts of the microtomed cell wall layers showed lower adhesion towards the hydrophobic tip at elevated temperatures.

Then, the heating stage was again set to 23 °C, the step motor was adjusted and the area of interest was traced by correcting the x- and y-position. At a temperature of 23 °C the areas close to the inner lumen surface, the whole cell wall layers and the middle lamella showed less adhesion towards the hydrophobic tip indicating an overall more hydrophilic character. This topography and adhesion imaging cycle (at 23 °C then 40 °C) was repeated three times (Fig. 3).

Areas close to the inner lumen surface constantly appeared in brighter color at 40 °C in the cyclic measurements. The alteration of the surrounding temperature led to a change of the adhesion forces in these areas, which are attributed to a comparably more hydrophobic surface characteristic. Additionally, the adhesion contrasts kept constant and no abrupt changes, which might appear if the tip breaks or is contaminated, were visible in these cyclic measurements. On microtomed unmodified cell wall areas, hydroxyl groups are predominant, which lead to constantly more hydrophilic surface properties. This can be explained by the general trend of CH_3_-terminated tip showing higher affinity towards hydrophobic/-CH_3_- chemical groups, and less affinity towards hydrophilic groups^21^.

These results indicate that the areas close to the lumen were grafted with PNIPAM chains as they clearly showed a stimuli-responsive behavior demonstrating the tuneable and reversible functionality of the introduced polymer at a cellular level. In addition, the position of thermo-responsive areas was revealed with high resolution and corresponded with the results from Raman microscopy. When comparing Figs. 2b and 3, thin areas close to the lumen of a transitionwood cell wall indicate the area of the introduced functionality in both characterization methods, CFM and Raman microscopy. Furthermore, also parts of the middle lamella showed a change in polarity only in the CFM maps. Hence, there is some evidence that also parts of the middle lamella showed a thermo-responsive behavior.

**Figure 4:**
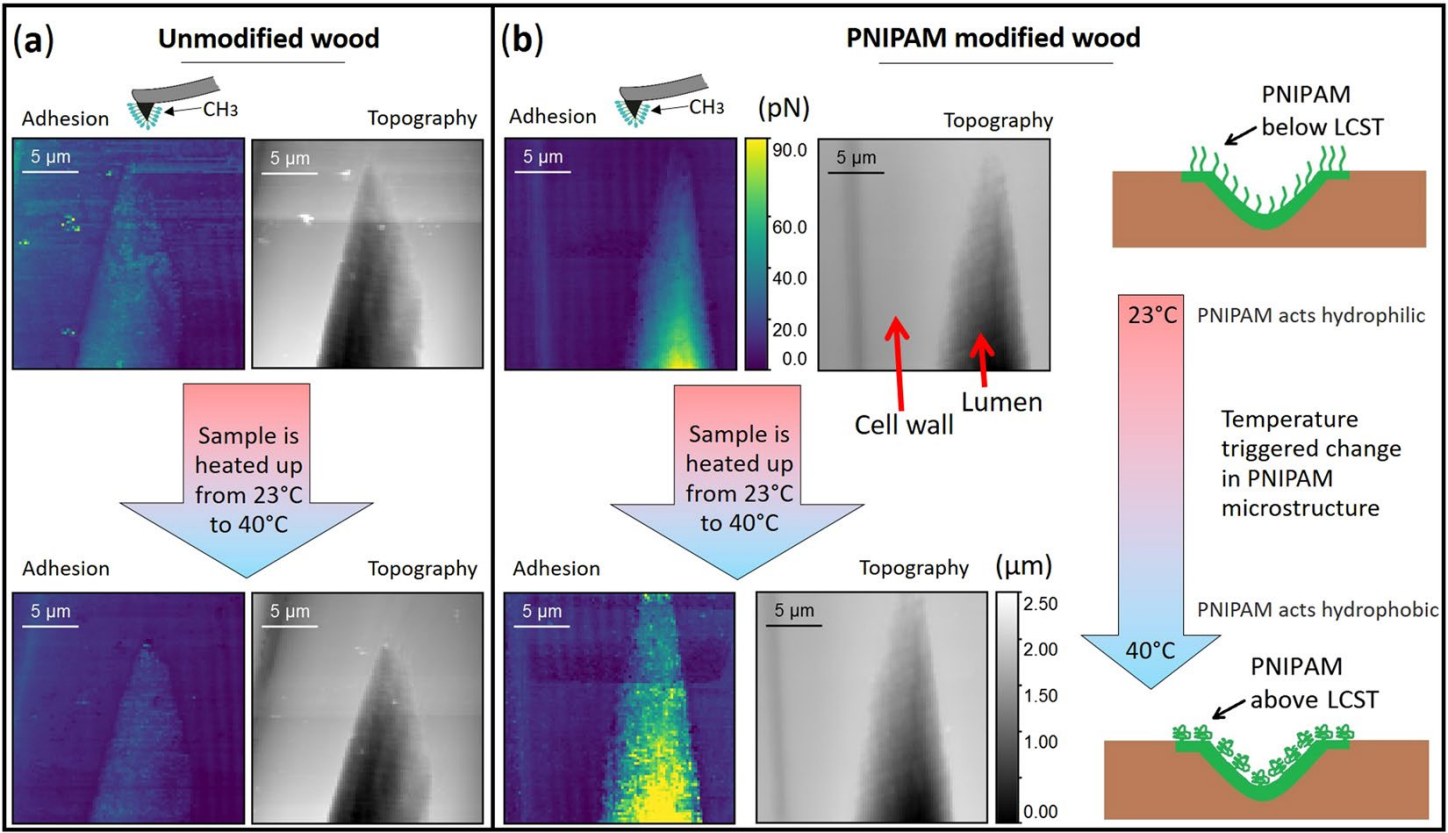
Representative CFM adhesion images of an unmodified (**a**) and a PNIPAM modified (**b**) wood cell wall and lumen, obtained with a CH_3_-terminated tip at different temperatures. Adhesion changes of the modified areas occur due to the hydrophobic collapse of the PNIPAM polymer chains above the LCST. The adhesion and topography scales are the same for each image at the unmodified and PNIPAM modified structures.

The same cyclic CFM measurements were conducted on unmodified wood samples, which served as reference surfaces. Adhesion maps of the radial section of a transitionwood cell wall were obtained with a CH_3_-terminated tip in water at 23 °C and 40 °C, respectively. Attention was given that the position and dimensions of the analyzed cell walls were similar at each analyzed sample. When looking at the CFM maps at both temperatures, 23 °C and 40 °C, the overall maps constantly showed lower adhesion forces towards the hydrophobic tip (Fig. 4). Contrary to a PNIPAM modified wood cell wall, only slight adhesion changes at cell wall areas close to the lumen or the middle lamella were visible at 40 °C. Additionally, the time required to stabilize the sample was longer and resulted in an extended total time to generate a whole cycle of CFM measurements. Some adhesion maps showed a wavelike pattern at thick cell wall areas but these patterns were not visible in the corresponding topography images (Fig. 4). According to the Nanoscope operating manual, that behavior is rather a result from the interference between the incident and reflected light from the samples surface, than a systematic adhesion effect of the tip on the samples surface.

Native, freshly microtomed wood surfaces essentially contain hydroxyl groups. Therefore, the swelling of the unmodified wood samples was much higher as there were more native hydroxyl groups of the wood structure available. On the contrary, the PNIPAM modified wood samples showed a less pronounced swelling behavior, because parts of the native hydroxyl groups were consumed in the modification, and not accessible for the surrounding water anymore. The slightly higher adhesion forces at native inner lumen surfaces of unmodified wood samples at 23 °C and 40 °C could be explained by the less polar character of the inner lumen surface compared microtomed cell wall areas^27^.

We observed a temperature driven change in polarity only at PNIPAM-modified areas. The change of PNIPAM from a hydrophilic to a hydrophobic character at temperatures above its LCST arises from its structural changes: below LCST the PNIPAM polymer chains are swollen and above ~32 °C the polymer is in a hydrophobic collapsed state^32^ which leads to a change in the probed adhesion values. Our results correlate well with various publications, in which the adhesion changes of PNIPAM-modified polymer brushes^15^ or microgel films^16–18^ were investigated by Scanning Probe Microscopy and showed switchable measured adhesion forces depending on the surrounding temperatures. Contrary to these experiments, our samples are naturally heterogeneous with height changes up to 4 µm in the scanning area and the wood structure swells in water in combination with expanding under temperature influences. Despite these given factors, we could visualize that specific parts of the wood matrix were functionalized and possess a thermo-responsive function. Besides, the degree of modification differed from cell wall to cell wall. This is related to the natural variability of wood materials (compare Figs. 3 and 4).

**Figure 5:**
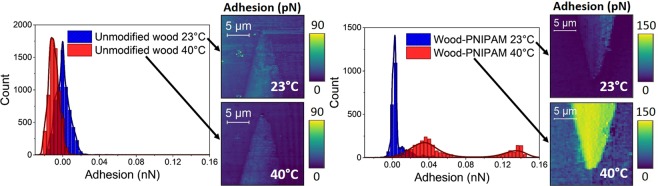
Distribution of the adhesion forces measured with a CH3-terminated tip at different temperatures of an unmodified wood sample (left histogram) and a modified wood sample (right histogram).

The adhesion force values corresponding to the modified wood structures of Fig. 3 and the unmodified wood structures of Fig. 4 were cropped to the same size in order to select and plot the exact same areas and plotted as histograms. The histograms underline the thermo-responsive behavior of functionalized wood cell walls when compared to an unmodified wood cell wall (Fig. 5). Note that we focused on the *change* in adhesion forces in this investigation. The distribution of the adhesion values of the unmodified sample at 23 °C showed a peak at 0 nN. Moreover, the distribution of the adhesion values of the PNIPAM-modified wood sample shows a plateau near 0 nN at 23 °C. At 40 °C there is a slight shift of the adhesion towards lower values when looking at the unmodified wood. All in all, the mean adhesion forces measured for these 3 configurations (unmodified/23 °C, unmodified/40 °C and modified/23 °C) are very low and do not show a high scattering. This is different for the fourth configuration – modified cell wall at 40 °C: the distribution of the adhesion forces measured on PNIPAM modified wood samples is much broader with two peaks at 0.036 nN and 0.136 nN. This distribution indicated the presence of a wider range of surface properties, compared to the narrow distribution of the unmodified samples. Therefore, there is some evidence of two distinct surface regions, which confirmed that parts of the analyzed wood cell wall area were functionalized. The applied characterization techniques did therefore not only reveal the position of thermo-responsive areas at the nano- and microscale level but also confirmed the functionality of the introduced polymer in aqueous media.

It was found that friction and adhesion forces increase linearly with applied loading forces^33^. Hence, the overall low adhesion forces originate from the low applied loading forces, which were chosen to prevent tip damage when performing long CFM measurements on rough surfaces. Adhesion force measurements with CH_3_-terminated tips on lignocellulosic fibers also resulted in low adhesion forces, which could be explained by the accessibility of the functional groups at the fibers surface^34^.

## Conclusions

In the present study, we demonstrated that CFM can be applied on complex wood surfaces. Our main findings are the followings:

- We could apply a method originally designed for flat surfaces in the field of nanotechnology on naturally inhomogeneous wood surfaces caused by the porous wood matrix. Measurements were performed in a liquid environment and CFM experiments were applied on wood structures at different temperatures. With proper sample preparation and scanning settings we were able to cope with the influence of wood swelling when conducting CFM measurements in water.
- Raman experiments confirmed the success of the *in-situ* polymerization and revealed the distribution of functionalized wood areas, with differences between early-, transition- and latewood cell walls. With CFM measurements we could assess the direct interaction of a hydrophobic tip on wood structures and analyze thermo-responsive properties.
- We could visualize the stimuli-responsive behavior of PNIPAM-modified wood at the cell wall level. The results indicate that CFM can be applied to locate and confirm introduced functional groups in a wood matrix with high resolution – a missing method in the field of wood material science.

## Methods

### Materials

All functionalization processes and CFM measurements were applied on blocks of spruce wood (*Picea abies*) with dimensions of 10 × 10 × 5 mm^3^ (radial x tangential x longitudinal). As a softwood, the structure of spruce wood is rather simple and its cellular components are mostly axially oriented tracheids and radial ray cells^3^. This structure offers a more constant and regular scaffold compared to hardwoods, and therefore offers a suitable matrix for the following functionalization and characterization approach.

All applied chemicals were purchased from Sigma-Aldrich (Darmstadt, Germany).

### Functionalization of wood

**Figure 6:**
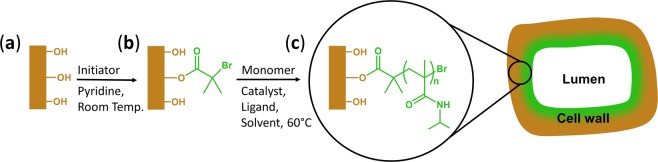
Modification procedure through a two-step surface-initiated Atom Transfer Radical Polymerization (ATRP) of the wood cell. (**a**) A native wood surface possesses mainly hydroxyl groups (brown color). (**b**) These hydroxyl groups are engaged in the esterification with BiBB (an α-bromoisobutyril bromide), yielding the wood macroinitiator used in the second functionalization step. (**c**) PNIPAM chains are growing from the macroinitiator, and the polymer is finally chemically bonded to the wood structure. The estimated area of the modified cell wall cross section is indicated with green color.

The grafting of PNIPAM chains inside the wood scaffold is a two-step process. In the first step, an Atom Transfer Radical Polymerization (ATRP) initiator is attached to wood through the esterification of hydroxyl groups from wood biopolymers. The obtained macroinitiator is then used in the second step, to start the ATRP of NIPAM monomers, yielding wood grafted with PNIPAM chains in specific areas (Fig. 6). The detailed functionalization process is reported in a previous publication^11^. Briefly, spruce wood blocks were oven dried, put into a flask and vacuum was applied. A solution of α-bromoisobutyril bromide (BiBB) in pyridine was prepared and slowly added to the flask. To calculate the amount of BiBB, we used a 0.5 molar equivalent with wood glucopyranose equivalents (MW = 162 g/mol). After stirring at room temperature, the esterified wood blocks were removed, washed with acetone and dried at 65 °C for 24 hours under vacuum. This modification yielded a solid macroinitiator for the next *in-situ* polymerization step. Two Schlenk flasks were prepared for the wood functionalization: the esterified wood blocks (Wood-Initiator) were placed in one flask with a gas inlet and a septum. The other flask contained a copper-based complex CuBr/N,N,N′,N′,N′′-pentamethyldiethylenetriamine (PMDETA), the monomer N-Isopropylacrylamide (NIPAM), and the solvent Dimethylformamid (DMF). After three freeze-pump-thaw cycles to degas it, the solution was transferred to the other flask using a cannula. The polymerization of NIPAM inside the wood blocks was run at 60 °C for 18 h under nitrogen atmosphere. As reported in the previous publication, the concentration of the reactants was set to [Monomer]:[Initiator]:[Catalyst]:[Ligand] = [NIPAM]/[Wood-initiator]/[CuBr]/[PMDETA] = 50:1:1:3. Subsequently, the polymerized wood samples were washed several times with acetone, EtOH or water. The polymerized wood blocks were then dried under vacuum at 65 °C for 24 hours. With these conditions, a weight percent gain of 75.5% was achieved.

### Sample preparation for Raman microscopy and chemical force microscopy

To perform CFM measurements in liquids, it is favorable to keep the dimensions of the wood sample as small as possible since the wood structure will swell during CFM measurements in water. On the other hand, they should provide the required thickness for microtoming and sample handling. The optimal sample dimensions were found to be 3 × 1.5 × 1.5 mm^3^ for our experimental set-up. Blocks with this sample size were cut out of unmodified and functionalized wood cubes with a razor blade. These specimen blocks were glued with a two-component epoxy resin (Uhu, Uhu plus sofortfest, Bühl/Baden, Germany) on AFM metal specimen discs. Plain cell wall surfaces were generated by an ultra-microtome (Ultracut-R, Leica, Wetzlar, Germany) with three different diamond knives (DiATOME, Nidau, Switzerland). Firstly, up to 500 nm sections were removed with a Trim 45 knife, followed by cutting off up to 1 µm with a Histo knife and finally removing 50 nm with an Ultra-AFM knife. The transverse section of the sample was microtomed for Raman microscopy, whereas the radial section was microtomed for CFM (Fig. 1 explains the difference between the two sections). The generated plain surfaces were subsequently analyzed with a light microscope (Zeiss Axioplan 2 Imaging, Jena, Germany) to choose proper cell wall areas for the further characterization steps.

### Raman measurements

On the transverse section, one annual ring was scanned using a confocal Raman microscope (alpha 300RA, WITec GmbH, Ulm, Germany) to visualize the PNIPAM modification. The microtomed wood blocks were placed in a fluid cell and immersed with deionized water 45 min before analysing to allow for sample stabilization. With a 785 nm laser (diode laser, CrystaLaser, Reno, NV, USA; 180 mW), a water immersion objective (Carl Zeiss, Germany, NA = 1), an optimized blazed grating (600 gmm^−1^, UHTS spectrometer, Witted Germany) and a deep depletion charge-coupled device camera (ANDOR, DU401A BR-DD), Raman spectra were acquired pixel by pixel (step size 0.33 µm) with an integration time of 0.083 s. The acquired spectral data were treated with a cosmic ray removal filter and chemical images calculated using the Sum-filter (position 1603 cm^−1^ for lignin, position 299 cm^−1^ for initiator and position 848 cm^−1^ for PNIPAM) in the WITec Project 4.1 Software. Average spectra of the chemically different areas are based on single Raman spectra of all red, green, blue pixels, respectively and were extracted based on intensity threshold filters applied on the band integration images. Images were generated by calculating the integrated area under the characteristic Raman bands in the spectra which range from 1553.7–1644.3 cm^−1^, 831.1–862.9 cm^−1^ and 280.7–319.3 cm^−1^. After baseline correction and smoothening of the data in a spectroscopy software (Spectragryph 1.2, Oberstdorf, Germany), the data was plotted with OriginPro 2016 (OriginLab, Northampton, MA, USA).

### Chemical force microscopy measurements

CFM measurements were performed with a Dimension Icon Scanning Probe Microscope (Nanoscope V, Bruker, Santa Barbara, CA, USA). Chemically sensitive maps were gained with CH_3_-terminated tips (ST-PNP-CH3, triangle shaped cantilever, NanoAndMore, Wetzlar, Germany), which were cleaned with ethanol and dried with a gentle steam of nitrogen before putting them onto the AFM fluid probe holder. The spring constants were determined by thermal tune (nominal spring constant = 0.32 N/m, resonance frequency = 67 kHz, half cone angles = 30–35°) and the deflection sensitivity was established on a sapphire test sample after the CFM measurements (n = 3). Thus, the CFM maps were acquired in Volts and converted into Newton after the measurements, so that the fragile tips were not damaged prior mapping. A new tip was used for each sample. A freshly microtomed sample was placed in the middle of the AFM fluid cell (Bruker, Santa Barbara, CA, USA), which was then put onto the implemented AFM heating stage (Lake Shore temperature controller, Westerville, Ohio, USA). The radial sections of the samples were investigated with CFM, since the radial profiles possess suitably smooth surfaces with no abrupt height changes, as opposed to the transverse section. Therefore, a starting point for scanning was chosen in air at the end of a cut-open cell wall, as shown in one of our previous studies^27^.

Subsequently, the fluid cell was filled with MilliQ water (18 MΩ, Labexchange, Burladingen, Germany), the temperature sensor of the heating stage was put inside the water to trace temperature changes and PeakForce QNM^©^ (Quantitative NanoMechanics, Bruker, Santa Barbara, CA, USA) measurements were performed. In additional tests, lateral forces were also analyzed in contact mode, but since long measurements were conducted on the rough wood surfaces, the tip did not endure measurement cycles at different temperature levels.

**Figure 7:**
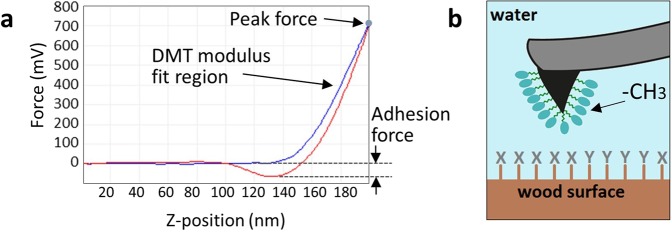
The Chemical force microscopy principle was applied to characterize the chemical properties of a wood cell wall. (**a**) The force-distance curve of the PeakForce QNM measurement displays the movement of the AFM tip in Z-position. The tip approaches the samples surface until a predefined peak force (blue line) and is attracted to a certain extent to the samples surface when the tip pulls away (red line), which is related to the adhesion forces between a (**b**) chemically modified tip and the (modified) wood surface.

The experiments were conducted in an aqueous environment to overcome capillary forces on the hygroscopic wood surface. Water was chosen as the surrounding medium so that the polymer can exhibit its thermo-responsive behavior and is not attracted by the tip reaching higher adhesion forces^21^. Force curves were collected in PeakForce QNM^©^ mode on the fly, simultaneously providing force-distance curves and height images. The adhesion force of the chemically modified tip on the samples surface was calculated from the force-distance curves and served as the key parameter to locate the functional groups in the present study (Fig. 7). These adhesion values were displayed as false color images at two temperatures (23 °C and 40 °C) and indicated the position and functionality of the introduced functional groups at the radial wood section. The interaction between the CH_3_-terminated tips and the (modified) wood surfaces resulted in contrasts in the CFM maps depending on the surface chemistry, morphology, mechanical properties and the surrounding media^33^. Minimal forces were applied to prevent tip damage scanning 128 pixels per line with a scan rate of 0.1 Hz. The lift height was set to 20–30 nm with a gain of 3–4 and the peak force amplitude was 100 nm.

Within the scope of this publication, two unmodified and two functionalized wood samples were investigated. Data analyzes were performed using OriginPro to plot histograms together with Kernel-smoothening, Nanoscope software and a Scanning Probe Microscopy analyzes tool (Gwyddion 2.49 freeware, Brno, Czech Republic).

## References

[1] Fratzl P, Weinkamer R (2007). Nature’s hierarchical materials. Prog. Mater Sci..

[2] Chinga-Carrasco G (2011). Cellulose fibres, nanofibrils and microfibrils: The morphological sequence of MFC components from a plant physiology and fibre technology point of view. Nanoscale Res. Lett..

[3] Wiedenhoeft, A. In *Handbook of Wood Chemistry and Wood Composites* (ed. Roger M. Rowell) Ch. 2, 9 (CRC Press, 2013).

[4] Berglund LA, Burgert I (2018). Bioinspired Wood Nanotechnology for Functional Materials. Adv. Mater..

[5] Sandberg, D., Kutnar, A. & Mantanis, G. Wood modification technologies - a review. *iForest - Biogeosciences and Forestry* **10**, 895, 10.3832ifor2380-010 (2017).

[6] Rowell RM (2006). Chemical modification of wood: A short review. Wood Mater. Sci. Eng..

[7] Petrič M (2013). Surface Modification of Wood. Reviews of Adhesion and Adhesives.

[8] Li Y, Vasileva E, Sychugov I, Popov S, Berglund L (2019). Optically Transparent Wood: Recent Progress, Opportunities, and Challenges. Adv. Opt. Mater..

[9] Yu Y (2012). Surface functionalization of bamboo with nanostructured ZnO. Wood Sci. Technol..

[10] Vitas S, Keplinger T, Reichholf N, Figi R, Cabane E (2018). Functional lignocellulosic material for the remediation of copper(II) ions from water: Towards the design of a wood filter. J. Hazard. Mater..

[11] Cabane E, Keplinger T, Künniger T, Merk V, Burgert I (2016). Functional lignocellulosic materials prepared by ATRP from a wood scaffold. Sci. Rep..

[12] Keplinger T (2016). Smart Hierarchical Bio-Based Materials by Formation of Stimuli-Responsive Hydrogels inside the Microporous Structure of Wood. Adv. Mater. Interfaces.

[13] Heskins M, Guillet JE (1968). Solution Properties of Poly(N-isopropylacrylamide) AU - Heskins, M. J. Macromol. Sci. Part A Pure Appl. Chem..

[14] Gierlinger N (2018). New insights into plant cell walls by vibrational microspectroscopy. Appl. Spectrosc. Rev..

[15] Svetushkina E, Puretskiy N, Ionov L, Stamm M, Synytska A (2011). A comparative study on switchable adhesion between thermoresponsive polymer brushes on flat and rough surfaces. Soft Matter.

[16] Aufderhorst-Roberts A (2018). Nanoscale mechanics of microgel particles. Nanoscale.

[17] Tagit O, Tomczak N, Vancso GJ (2008). Probing the Morphology and Nanoscale Mechanics of Single Poly (N-isopropylacrylamide) Microgels Across the Lower- Critical‐Solution Temperature by Atomic Force Microscopy. Small.

[18] Schmidt S (2010). Adhesion and mechanical properties of PNIPAM microgel films and their potential use as switchable cell culture substrates. Adv. Funct. Mater..

[19] Frisbie CD, Rozsnyai LF, Noy A, Wrighton MS, Lieber CM (1994). Functional group imaging by chemical force microscopy. Science.

[20] Noy A, Frisbie C, F. Rozsnyai L, Wrighton M, Lieber C (1995). Chemical Force Microscopy: Exploiting Chemically-Modified Tips To Quantify Adhesion, Friction, and Functional Group Distributions in Molecular Assemblies. J. Am. Chem. Soc..

[21] Noy A, Vezenov DV, Lieber CM (1997). CHEMICAL FORCE MICROSCOPY. Annu. Rev. Mater. Sci..

[22] Noy A (2006). Chemical force microscopy of chemical and biological interactions. Surf. Interface Anal..

[23] Fu W, Carbrello C, Wu X, Zhang W (2017). Visualizing and quantifying the nanoscale hydrophobicity and chemical distribution of surface modified polyethersulfone (PES) membranes. Nanoscale.

[24] Beaussart A (2014). Chemical force microscopy of stimuli-responsive adhesive copolymers. Nanoscale.

[25] Effah B, Raatz K, Reenen AV, Meincken M (2017). Chemical force microscopy analysis of wood-plastic composites produced from different wood species and compatibilizers. Wood Fiber Sci..

[26] Colson J (2018). Adhesion properties of regenerated lignocellulosic fibres towards poly (lactic acid) microspheres assessed by colloidal probe technique. J. Colloid Interface Sci..

[27] Frybort S, Obersriebnig M, Müller U, Gindl-Altmutter W, Konnerth J (2014). Variability in surface polarity of wood by means of AFM adhesion force mapping. Colloids Surf., A.

[28] Ito T, Grabowska I, Ibrahim S (2010). Chemical-force microscopy for materials characterization. Trends Anal. Chem..

[29] Vidiella del Blanco M, Gomez V, Keplinger T, Cabane E, Morales LFG (2019). Solvent-Controlled Spatial Distribution of SI-AGET-ATRP Grafted Polymers in Lignocellulosic Materials. Biomacromolecules.

[30] Fengel, D. & Wegener, G. *Wood Chemistry, Ultrasture, Reactions*. (Kessel Verlag, 2003).

[31] Niemz, P. & Sonderegger, W. *Holzphysik: Physik des Holzes und der Holzwerkstoffe*. (Carl Hanser Verlag GmbH Co KG, 2017).

[32] Kubota K, Fujishige S, Ando I (1990). Solution Properties of Poly(N-isopropylacrylamide) in Water. Polym. J..

[33] Vezenov, D. V., Noy, A. & Lieber, C. M. In *Handbook of Molecular Force Spectroscopy*. (ed. Aleksandr Noy) Ch. 4, 123-141 (Springer, 2008).

[34] Bastidas JC (2005). Chemical force microscopy of cellulosic fibers. Carbohydr. Polym..

